# Photon Drag Currents and Terahertz Generation in α-Sn/Ge Quantum Wells

**DOI:** 10.3390/nano12172892

**Published:** 2022-08-23

**Authors:** Binglei Zhang, Yi Luo, Yang Liu, Valerii N. Trukhin, Ilia A. Mustafin, Prokhor A. Alekseev, Bogdan R. Borodin, Ilya A. Eliseev, Fatemah H. Alkallas, Amira Ben Gouider Trabelsi, Anna Kusmartseva, Fedor V. Kusmartsev

**Affiliations:** 1 Microsystem and Terahertz Research Center, Chengdu 610200, China; 2 Ioffe Physical Technical Institute, Polytekhnicheskaya St., 26, St. Petersburg 194021, Russia; 3 Department of Physics, College of Science, Princess Nourah bint Abdulrahman University, P.O. Box 84428, Riyadh 11671, Saudi Arabia; 4 Department of Physics, Loughborough University, Loughborough LE11 3TU, UK; 5 Department of Physics, Khalifa University of Science and Technology, Abu Dhabi 127788, United Arab Emirates

**Keywords:** topological insulator, THz radiation, gray tin, quantum well

## Abstract

We have fabricated α-Sn/Ge quantum well heterostructures by sandwiching nano-films of α-Sn between Ge nanolayers. The samples were grown via e-beam deposition and characterized by Raman spectroscopy, atomic force microscopy, temperature dependence of electrical resistivity and THz time-resolved spectroscopy. We have established the presence of α-Sn phase in the polycrystalline layers together with a high electron mobility μ = 2500 ± 100 cm^2^ V^−1^ s^−1^. Here, the temperature behavior of the resistivity in a magnetic field is distinct from the semiconducting films and three-dimensional Dirac semimetals, which is consistent with the presence of linear two-dimensional electronic dispersion arising from the mutually inverted band structure at the α-Sn/Ge interface. As a result, the α-Sn/Ge interfaces of the quantum wells have topologically non-trivial electronic states. From THz time-resolved spectroscopy, we have discovered unusual photocurrent and THz radiation generation. The mechanisms for this process are significantly different from ambipolar diffusion currents that are responsible for THz generation in semiconducting thin films, e.g., Ge. Moreover, the THz generation in α-Sn/Ge quantum wells is almost an order of magnitude greater than that found in Ge. The substantial strength of the THz radiation emission and its polarization dependence may be explained by the photon drag current. The large amplitude of this current is a clear signature of the formation of conducting channels with high electron mobility, which are topologically protected.

## 1. Introduction

The discovery of topological insulators (TI) [1] and Weyl semimetals [2] led to a “topological revolution” in materials science. These types of materials open an avenue for the creation of novel photodetectors with significantly enhanced sensitivity compared to those based on conventional metals, insulators, and semiconductors. Indeed, topological materials have Weyl cone-like electronic structures uniquely characterized by the Berry curvature. There, photocurrent is greatly enhanced and may be used to fabricate photodetectors with extreme sensitivity, capable of single-photon detection when the excitation takes place in the vicinity of Weyl nodes, where the Berry curvature diverges [3]. Quite recently, it was shown that multilayer structures TaAs, TaP, NbAs, NbP, Bi_1–x_Sb_x_ are Weyl semimetals [4,5,6,7]. Over the past few years, this area has attracted increasing interest from the theoretical and experimental communities. General progress in theoretical phenomenologies, new material development and novel device fabrication has been summarized in a recent detailed review on Weyl semimetals [8]. Despite a surge in research activity, there have been relatively few experimental studies on the optical properties of these materials. For example, circular photocurrent and photovoltaic effects have been observed in TaAs at an excitation photon energy ħω ≈ 2.38 eV [9] and under a range of excitation frequencies, including illumination with a CO_2_ laser [10,11]. Most recently, a trend has emerged focusing on the fabrication of topological materials by combining traditional elements into multilayers and utilizing time reversal symmetry breaking and band inversion. Notably, breakthroughs have been achieved in studies of InAs/GaSb bilayer quantum wells (QW), demonstrating quantum spin hall insulators (QSHI) phases and unique edge state transports [12]. Similarly, research conducted on InAs/GaSb/InAs three-layer QWs has shown interband optical transition and activity in the THz regime [13]. Therefore, the extent of the topological materials based on multilayer superlattices and the evolution of their optical properties in relation to the topological phases is a rapidly expanding, timely and promising field.

Here, we propose the creation of topological materials, including two and three dimensional topological insulators (2DTI and 3DTI), as well as Weyl semimetals, by exploiting the combination of traditional materials, such as Sn and Ge films, in a multilayer heterostructure. The premise focuses on the fabrication of systems with mutually inverted band structures. Theoretical works have shown that such materials demonstrate gapless states with linear carrier dispersion [14,15]. Specifically, the appearance of a metallic phase with a linear electronic spectrum was predicted at the interface of a heterojunction between two Ge and α-Sn semiconductors due to the appearance of a mutually inverted band structure—where the valence band of Ge is transformed to the same irreducible representation of the symmetry group as the conduction band of Sn, and vice versa.

Recently, a-Sn film has been shown to behave as a topological Dirac semimetal with unusual properties. For example, α-Sn films were successfully grown on an InSb(111) substrate [16]. Further research demonstrated that extremely high quality α-Sn films with record hard mobilities of 30,000 cm^2^ V^−1^ s^−1^ could be obtained on InSb(001) substrates [17]. α-Sn films are extremely sensitive to strain and can be tuned to transition from a topological Dirac semimetal (TDS) to a two-dimensional topological insulator (2DTI) by varying their thickness [16,17]. It was also discovered that a system of a-Sn/CdTe quantum well (QW) undergoes a phase transformation when the well width increases above a critical value of 8 nm [18]. In Ref. [18] it was shown that when the a-Sn/CdTe QW width exceeds the critical width, the material behaves as a 2DTI, whereas below this value, it acts as a narrow-band semiconductor. The paradigm of our present work is to create a hybrid topological material in the form of a superlattice, QWs or a heterostructure, where the symmetry of inversion or time reversal symmetry is broken, leading to the formation of Weyl cones.

Bulk Sn is present as a metastable white tin metal phase (β-Sn) at room temperature. β-Sn undergoes a gradual transformation into the gray tin phase (α-Sn) when the temperature is decreased to 286.4 K. In the case of Sn thin films, the temperature of this phase transition varies with volume, growth conditions and the substrates on which the synthesis is carried out. For example, for Sn films grown on an InAs substrate, the temperature for the β—to—α transformation is generally higher than room temperature (300 K) and is strongly dependent on the thickness of the Sn film and on the orientation of the substrate [19]. Notably, GeSn alloys, even at low Ge concentrations, form a diamond crystal structure similar to α-Sn above room temperature [20]. Thus, it is expected that by growing Sn nanolayers on a Ge surface, it would be possible to stabilise α-Sn even at high temperatures (~300 K). In this manner, by sandwiching one Sn nanolayer between two Ge layers, one may obtain an α-Sn and Ge quantum well. The number of sandwiched Sn nanolayers will determine the number of quantum wells in the heterostructure. Our main aims are to fabricate single and multiple quantum well nanostructures based on nanometer Ge and α-Sn layers and to study their optical and electronic properties by studying the photocurrent both by contact method and by generating terahertz radiation using femtosecond optical pulses.

## 2. Results and Discussion

**Figure 1:**
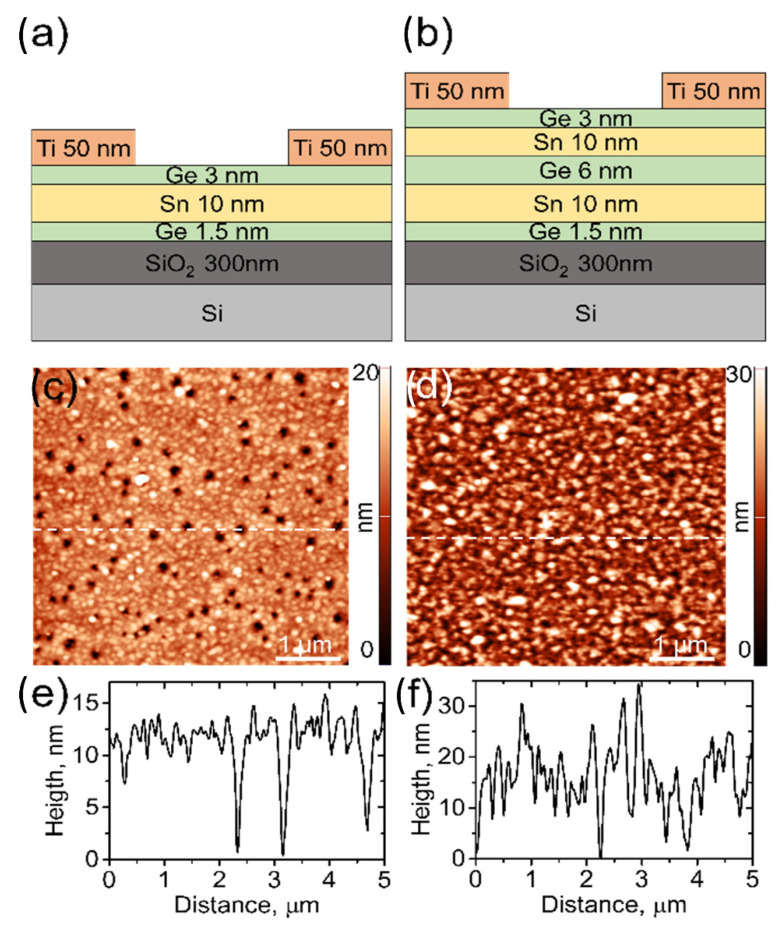
Schematic cross section of the investigated α-Sn/Ge quantum wells heterostructures. (**a**,**b**) Single (**left**) and double (**right**) α-Sn/Ge quantum wells were composed of Ge and α-Sn layers nanolayers, grown on 300 nm thick SiO_2_ surface on a Si substrate. (**c**,**d**) AFM topography images and the corresponding linescan topography profiles (**e**,**f**) of the investigated single and double α-Sn/Ge quantum wells heterostructures. Topography profiles were taken from the regions marked by white dashed lines in (**c**,**d**).

The investigated samples consisted of stacked Ge and α-Sn layers with varying nanometer thicknesses in a heterostructure grown on a 300 nm thick SiO_2_ surface deposited on a silicon (Si) substrate. The layer thickness ranged between 1.5 to 10 nm. All nanofilms were grown via the electron beam deposition method. For the photocurrent measurement of 50 nm thick titanium (Ti), electrodes were deposited onto the surface of the sample in a specific geometry through a rigid Si mask. The mask was laser cut from a 500 nm thick Si waver and corresponded to a linear configuration of four square 1 mm × 1 mm sized windows separated by 2 mm. The Si mask was brought into direct contact with the sample surface prior to Ti deposition. The schematic cross sections of the Ge and α-Sn layer samples with Ti electrodes are shown in Figure 1a,b. Single and double α-Sn and Ge quantum wells with overall thicknesses of 14.5 nm and 30.5 nm are depicted in Figure 1a,b, respectively. The thickness of α-Sn layer was 10 nm in both cases, while the Ge layers ranged in thickness from 1.5 nm–6 nm, depending on their location in the heterostructure. Electrical connections were made by gluing gold (Au) wires 25 μm in diameter using silver (Ag) and reinforcing them mechanically using an insulating resin. Alternatively, point-contact probes pressed onto Ti electrodes were used for electrical measurements.

Figure 1c,d show atomic force microscopy (AFM) topography images of the single quantum well and double quantum well, respectively. Corresponding linescan profiles are presented in Figure 1e,f. From the images, the polycrystalline structure of the layers is clearly seen. For the single quantum well structure, 8–14 nm deep pits are observed. Outside the pits, the surface has a roughness with a root mean square (RMS) of 1.2 nm. The surface of the double quantum well structure contains fewer pits, 10–15 nm deep; however, the RMS roughness is increased to 7 nm.

**Figure 2:**
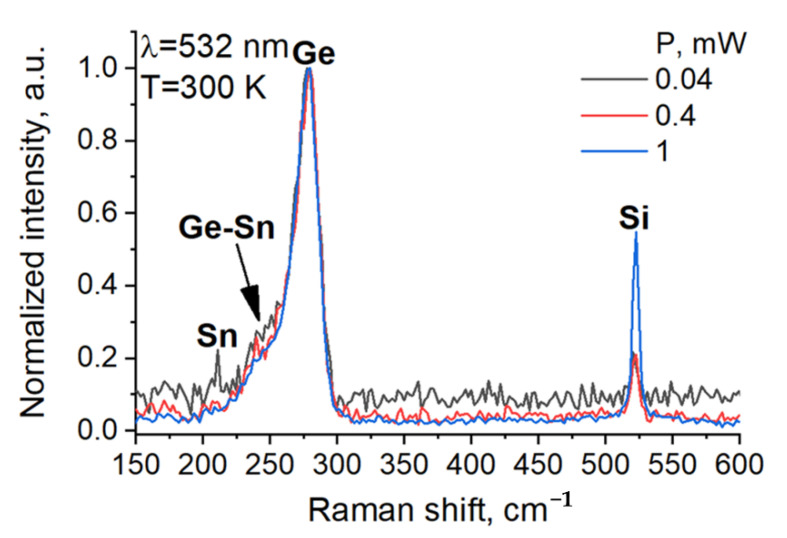
Raman spectra of a double α-Sn/Ge quantum well heterostructure. The studies were carried out at 40 μW (black line), 0.4 mW (red line) and 1 mW (blue line) laser power. The spectra show peaks corresponding to the Si substrate (521 cm^−1^), the nanocrystalline Ge (277.7 ± 0.2 cm^−1^) and the Ge–Sn shoulder mode (251 ± 3 cm^−1^). The α-Sn mode (210 ± 1 cm^−1^) is only visible at the lower laser power of 40 μW and disappears with heating.

The composition of the α-Sn/Ge quantum well heterostructures was confirmed using Raman spectroscopy at room temperature. Raman spectroscopy studies were performed in a “backscattering” geometry on a Horiba Jobin-Yvon T64000 spectrometer equipped with a confocal optical microscope and a YAG: Nd laser (λ = 532 nm) laser. During measurements, the laser spot was focused on an area with a diameter of ~1 μm using a 100× objective (NA = 0.9) and optical pumping was used to adjust the laser power in a range of 0.04–1 mW. An increase in power should lead to heating of the sample and consequently to an α—to—β phase transition [19]. It should be noted that at pump power of 2 mW, the film is locally destroyed (burned out). Figure 2 shows the Raman spectra measured at a power of 40 μW (black line), 0.4 mW (red line), and 1 mW (blue line). The spectra are normalized to the maximum intensity of the Ge mode. All spectra contain peaks corresponding to the Si substrate (521 cm^−1^), the nanocrystalline bulk Ge (277.7 ± 0.2 cm^−1^) [21], and the Ge—Sn shoulder mode (251 ± 3 cm^−1^) often observed in GeSn alloys [22]. Interestingly, at the lowest laser power of 40 μW an additional peak is present at 210 ± 1 cm^−1^ (see Figure 2 (black line)). This new peak disappears as the laser power is increased. The effect is reversible. The observed disappearance of the 210 cm^−1^ peak may be attributed to an α—to—β Sn phase transition as the sample is heated up locally with increased pumping power. Thus, the presence of the 210 cm^−1^ mode and its temperature dependence can be taken as evidence for the formation of α-Sn in the heterostructure. Similar behavior has been observed in published literature [19]. It should be noted that the frequency of the α-Sn mode (197 cm^−1^) reported in Ref. [19] is lower than the frequency of the α-Sn mode detected in the present work (210 cm^−1^). The difference may be explained by a change of substrate as well as substrate-induced elastic strain, which is more prominent here due to nanofilm thickness. Additionally, the Raman results indicate the formation of a Ge-Sn compound, which may be synthesized on the initial Ge layer (1.5 nm) prior to the growth of α-Sn.

**Figure 3:**
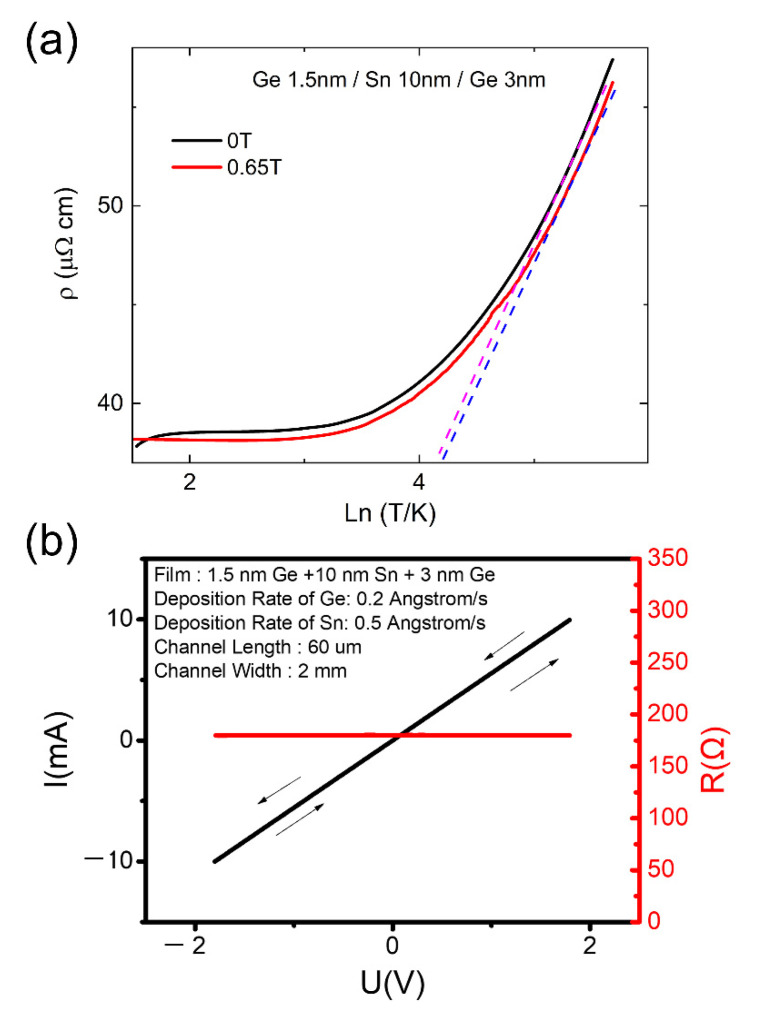
(**a**) Electrical resistivity *ρ_xx_* as a function of the logarithm of temperature in a single α-Sn/Ge quantum well heterostructure in 0T (black line) and 0.65T (red line). The temperature dependence of *ρ_xx_* does not manifest any step-like anomalies indicative of a α—to—β Sn phase transition across the investigated temperature range. The resistivity behavior shows a logarithmic temperature dependence >200 K which may be consistent with the Altshuler–Aronov (AA) regime. By comparing the zero and applied magnetic field resistivities at a fixed temperature the charge carrier mobility μ can be extracted. (**b**) IV characterization curve for a single α-Sn/Ge quantum well heterostructure in 0 T, signifying the presence of the ohmic regime in the system.

To further corroborate the presence of α-Sn and to determine its charge carrier mobility μ, electrical resistivity measurements were performed on a single α-Sn/Ge quantum well heterostructure across a temperature range of 4.7–300 K both in zero (0 T) and applied (0.67 T) magnetic fields, see Figure 3a. Electrical resistivity was measured using a standard linear 4-probe technique with DC current. The electrical drift bias was eliminated by reversing the current direction during the measurements. The base temperature of 4.7 K was obtained using a Sumitomo pulsed tube cryostat, and the magnetic fields were generated using a permanent disc-shaped magnet. The value of the magnetic field was determined using an external Hall probe at room temperature. The magnetic field was varied by adjusting the distance between the magnet and the sample. The study was carried out both while decreasing and increasing the temperature, and no thermal hysteresis was seen. Nominally, the α—to—β Sn phase transition would lead to a step like anomaly in the resistivity with thermal hysteresis but this was not observed. The temperature dependence of the resistivity remains monotonic and without step-like anomalies throughout the investigated temperature range. This further verifies that our fabricated α-Sn/Ge quantum well heterostructures contain α-Sn at room temperature.

Additionally, the in-plane resistivity *ρ_xx_* for the α-Sn/Ge quantum well heterostructure demonstrates a metallic behavior across the entire temperature range (both at 0 T and at 0.65 T). While conventionally bulk α-Sn resistivity is expected to follow semiconducting temperature dependence above 200 K [23], the behavior may manifest differently on the nanometer scale in α-Sn thin films. Moreover, the behavior also differs from that recently observed in the α-Sn/InSb (001) films acting as three-dimensional Dirac semimetals [24]. In-depth analysis of the electrical resistivity *ρ_xx_* at high temperatures (>200 K) reveals an Altshuler–Aronov (AA) regime, where electron–electron interactions (EEI) dominate the scattering properties. This behavior is notably signified by a logarithmic T dependence in the electrical resistivity described by the equation:

(1) $$\rho_{xx}=\rho_{0}\left[1+A\frac{\rho_{0}e^{2}}{2\pi^{2}\hbar }\left(\mu^{2}B^{2}-1\right)\ln\left(\frac{k_{B}\tau T}{\hbar }\right)\right]$$

where $\rho_{0}$ is the residual resistivity, A≤1 is a constant, e is the electron charge, T is the temperature, μ is the charge carrier mobility, B is the magnetic field and τ is the transport momentum relaxation time [25]. The logarithmic regime, as given by Equation (1), is highlighted with the purple and blue dashed lines for the zero-field (*B* = 0 T) and in-field (*B* = 0.65 T) measurements, respectively, see Figure 3a. Thus, it becomes possible to extract the charge carrier mobility μ and the relaxation time τ by comparing the zero-field and in-field resistivities as described by Equation (1) directly. No other input parameters are needed, and it is not necessary to know the value of the constant A. In the present case for a single α-Sn/Ge quantum well heterostructure, the mobility was calculated as $\mu =2500\pm 100\;{\mathrm{cm}}^{2}{\;V}^{-1}\;s^{-1}$ and the relaxation time was estimated as τ=2600±100 fs. The obtained mobility is comparable with the values for the InSb/α-Sn/AlOx 3DTI, where $\mu =3180\;{\mathrm{cm}}^{2}{\;V}^{-1}{\;s}^{-1}$ [26]. IV characterization curves signify that α-Sn/Ge quantum well heterostructures are in the ohmic regime, see Figure 3b. No hysteresis behavior was observed as the direction of the current was reversed. The IV response was measured using a 6221 Keithley current source and a 2182 Keithley nanovoltmeter, covering the ranges −10 mA to 10 mA and −2 V to 2 V for current and voltage, respectively. Note that the AA regime is absent in metallic Sn. Furthermore, any contributions from disorder effects (barring those from magnetic impurities) are invariant in an applied magnetic field, or provide positive magnetoresistance. When some metallic droplets are separated, e.g., droplets of β-Sn in α-Sn, there arises a large positive extraordinary magnetoresistance. See Refs. [27,28] for detailed discussions on these issues. Note that here we observe slight negative magnetoresistances, see Figure 3a. Similarly, the presence of disorder leads to very low mobilities, which is in contrast to what we observe. Thus, the combined results of logarithmic temperature dependence terms in the electrical resistivity, negative magnetoresistances and high mobilities suggest that the reason for the observed metallicity in the α-Sn/Ge quantum well heterostructures may be topological in nature.

The optical and THz generation properties of the α-Sn/Ge quantum well heterostructures were probed using THz time-resolved spectroscopy. During the optical studies, the samples were excited by ultrashort femtosecond laser pulses, and the waveform of the generated THz pulses was recorded and analyzed. As sources of optical radiation, two types of Ti:Sapphire lasers with diode pumping were used; specifically, MaiTai—for ~100 fs pulses in the wavelength range 710–950 nm at a repetition frequency of 80 MHz, average optical power ~1.5 W; Synergy—for radiation with a wavelength of ~800 nm and a pulse duration of ~15 fs at a repetition frequency of 76 MHz, average optical power ~0.5 W. Registration of the generated photocurrent by the contact method was measured by the Lock-in Amplifier Signal Recovery 7265 (in current mode) between the Ti electrodes on the sample surface, as well as the registration of THz radiation generated by photocurrents by THz time-domain spectroscopy.

**Figure 4:**
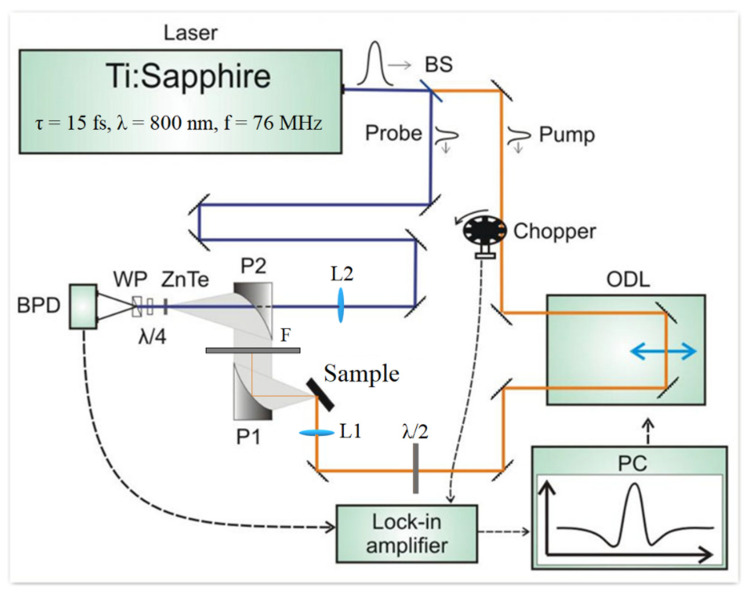
The THz spectrometer setup: femtosecond laser Synergy—pulse duration ~15 fs; P1 and P2—off-axis paraboloids—parabolic mirrors; WP—Wollaston prism; BPD—balance photodetector; λ/4—quarter-wave plate; ZnTe—non-linear optical crystal; ODL—optical delay line; Chopper—light modulator; Lock-in amplifier—Signal Recovery 7265 (current mode); THz emitter—sample; BS—light splitter (2% for probe and 98% for pump); F is a filter of the transmitted THz radiation, removing IR radiation tail; L1 and L2 are lenses focusing the optical radiation; λ/2—half-wave plate; PC—personal computer. The THz generated current pulse is analyzed in a specially designed LabView program.

The schematic diagram of the experimental setup for THz generation detection is shown in Figure 4, outlining the key steps in the measurement process. The main optical pulse of linearly polarized radiation (from the Ti:Sapphire laser) is divided into pump and probe pulses by means of the light splitting plate (BS). The pump pulse is mechanically modulated at a ~1 kHz frequency by the chopper. Modulation of the optical pumping is used to reduce the noise in the signal detection. The modulated pump pulse then passes through the optical delay line (ODL) and is focused on a selected area of the sample with an angle of incidence of 45 degrees. As a result of the excitation of the sample by the pump pulse, coherent THz radiation is generated, which is collected by gold sputtered parabolic mirrors (P1 and P2) and directed to the non-linear optical crystal (ZnTe). The probe pulse is sent directly to the ZnTe crystal through a hole in P2. The interaction between the THz radiation and the pump pulse in the ZnTe crystal induces birefringence and causes a change in light polarization from linear to elliptical, known as the Pockels effect. (Without the THz field the optical pulse passes through ZnTe unchanged). The change in light polarization is detected by an optical circuit including a λ/4 quarter-wave plate, a Wollaston prism (WP), and a photodiode balance detector (BPD). For a linearly polarized incident signal, the λ/4 quarter-wave plate adds circular polarization, and the WP splits the signal into two mutually perpendicularly polarized pulses arriving at the BPD. Thus, in the case of linearly polarized radiation, the illumination intensities detected by the two photodiodes in the BPD are the same. In the presence of the THz field, the light entering the λ/4 quarter-wave plate is elliptically polarized. Therefore, the photodiodes in the BPD detect different intensities, resulting in an output signal. The output signal from the BPD is fed into the lock-in amplifier (Signal Recovery 7265) synchronized with the modulated pump pulse, which allows the separation of the THz signal. The data from the lock-in amplifier is analyzed in a specially programmed LabView environment on the PC, which also controls the movement of the ODL to introduce further time delay between the pump and probe pulses. Thus, within one setup, it is possible to resolve the time dependence of the THz field by obtaining the relationship between the BPD output signal and the time delay between the pump and probe pulses. The THz spectrometer setup is designed to operate at room temperature. During the experiment, the Ge/Sn samples have been fixed to a holder made from copper (Cu). Cu has extremely high thermal conductivity, so the temperature was considered to be constant throughout the experiment and no temperature controller was used. All measurements were conducted at room temperature. Recently, we have also made the same THz measurements at liquid nitrogen temperatures. The results are qualitatively the same, while the efficiency of the THz radiation emission has been increased.

**Figure 5:**
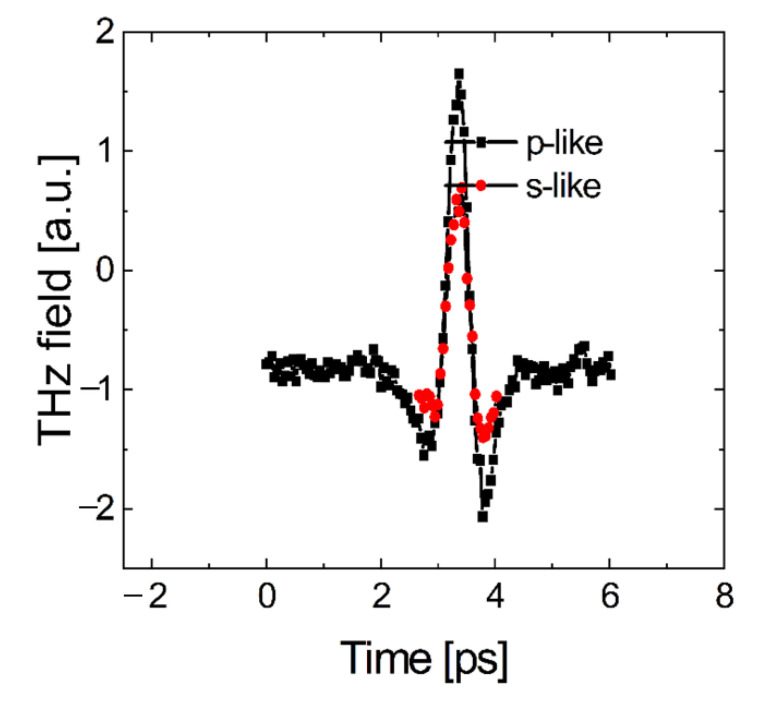
THz pulse waveform generated by a Ge film on a SiO_2_/Si substrate with 90 mW average optical power. The responses to TM (p-like) and TE (s-like) polarized light are shown in black and red, respectively. The THz pulses have the same phase in both polarizations, but the amplitude is lower for TE excitation compared with TM.

Initially, we have studied the generation of THz radiation in thin films of Ge (~50 nm thick) deposited on a SiO_2_/Si substrate. The Ge samples were excited with two different light polarizations: TM(p-like) and TE(s-like). The results are summarized in Figure 5, where the THz waveforms produced by TM and TE radiation are shown in black and red, respectively. Interestingly, the THz pulses generated by TM and TE light have the same phase. Here, the phase of the electric field of the THz pulse corresponds to the movement of electrons in the same direction as the vector component of light lying in the plane of the sample. In other words, the direction of the electric vector of the THz field remains the same when the polarization of incident light is changed. The amplitude of the THz field is smaller for TE polarization compared with TM. The phase calibration was performed using a bulk InAs semiconductor crystal. Based on the results, we postulate that the mechanism for THz generation in Ge thin films may be associated with ambipolar diffusion current of nonequilibrium charge carriers generated during interband transitions in Ge, similar to those found in semiconductors such as InAs [29].

**Figure 6:**
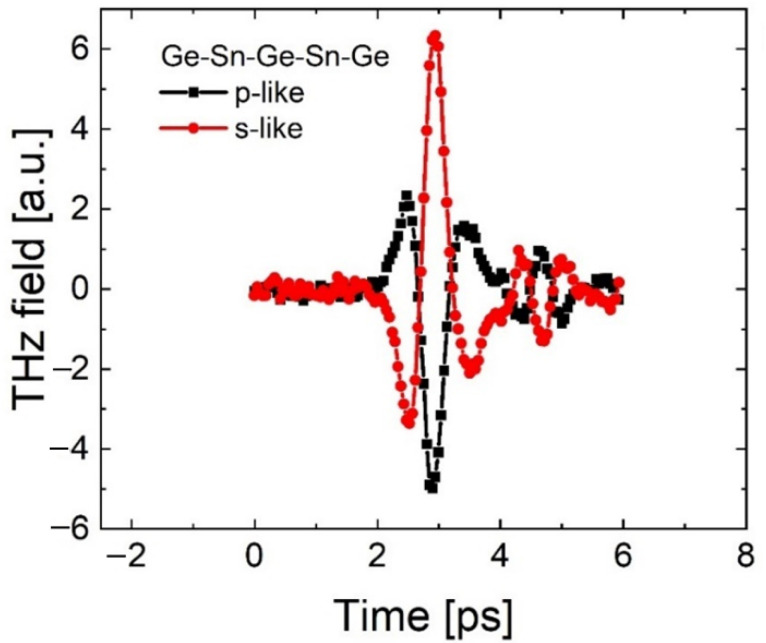
THz pulse waveforms generated by a double α-Sn/Ge quantum well heterostructure with 100 mW average optical power. The responses to TM (p-like) and TE (s-like) polarized light are shown in black and red, respectively. Notably, when the polarization of the excitation light changes from TM to TE, the generated THz pulses have the opposite phase, which indicates that the electric vector of the THz field changes direction.

Interestingly, studies of THz generation in samples containing α-Sn/Ge quantum wells lead to markedly different results, see Figure 6, where the THz waveforms produced by TM and TE radiation are shown in black and red, respectively. Specifically, the THz pulses generated by TM and TE light have the opposite phase. This means that when the polarization of incident light changes from TM to TE, the direction of the THz electric field vector reverses. In other words, when the single and double α-Sn/Ge quantum wells are excited by TM polarized pulses to generate a THz field, the electrons begin moving in the opposite direction as the vector component of light lying in the plane of the sample—a result in sharp contrast with previous experiments on Ge thin films. Our findings imply that the mechanism for THz generation in single and double α-Sn/Ge quantum wells samples would be significantly different to those found in conventional semiconductors.

**Figure 7:**
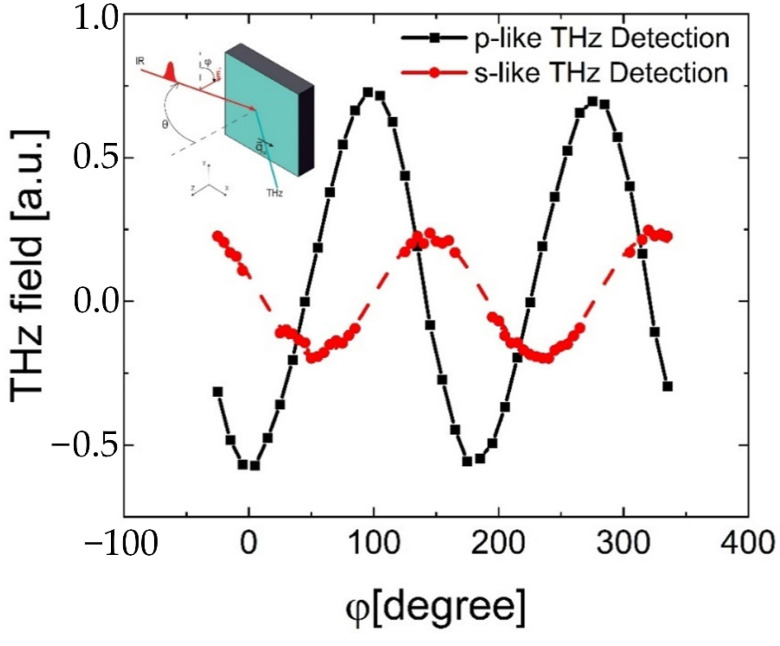
The dependence of the amplitude of the THz pulses generated by a double α-Sn/Ge quantum wells with 100 mW average optical power on the angle of rotation of light polarization φ. The responses to TM (p-like) and TE (s-like) polarized light are shown in black and red, respectively. The angle of light incidence on the sample was 45 degrees. The angle between the direction of the THz electric field and the normal to the surface of the sample was 90 degrees. The amplitude maxima for the THz field in TE polarization were found at 0, 90, 180 and 270 degrees. While the amplitude maxima for the THz field in TM polarization were found at 45, 135, 225 and 315 degrees.

Additional optical experiments on single and double α-Sn/Ge quantum wells samples have revealed that the amplitude of the generated THz fields demonstrates substantial dependence on the angle of rotation of the polarization of incident light (see, Figure 7). Specifically, it was shown that the dependence of the electric field strength of the THz pulse (or its amplitude) on the angle of rotation of the light polarization is sinusoidal. For the TM (p-like) induced THz field, it is proportional to cos2φ, with amplitude maxima occurring at 0, 90, 180 and 270 degrees. While for the TE (s-like) induced THz field, the amplitude maxima vary as sin2φ—with antinodes found at 45, 135, 225 and 315 degrees (see Figure 7). The dependence of the maximum amplitude of the THz pulse on the intensity of the incident light was linear.

Studies of photocurrent generation by the contact method on single and double α-Sn/Ge quantum well samples mirrored the trends found in all other experiments. In addition, a sign inversion of the photocurrent was observed when the angle of light incidence was changed from 45° to −45°. Notably, Ge thin film samples showed a significant decrease in the efficiency of THz generation and, accordingly, a substantially lower photocurrent compared to samples containing α-Sn/Ge quantum wells.

The origin for the experimentally observed dependences of the photocurrent in single and double α-Sn/Ge quantum well heterostructures on light angle of polarization, wave vector and intensity may be explained by photon drag current mechanism. In general, the photon drag current is determined by the fourth-order tensor and can be written in the following form [30]:

(2) $$j_{i}{=\chi }_{\mathrm{ijkl}}E_{j}E_{k}q_{l}$$

where E_j_ is the j component of the electric field of the light wave, q_l_—l is the component of the wave vector of light.

For an isotropic medium, the photon drag current will be determined by two independent components of this nonlinear tensor. For the experimental geometry shown in the inset in Figure 4, the projections of the current on the coordinate axes will be determined by:

(3) $$j_{x}{=\sin\theta (a+2\mathrm{bcos}}^{2}{\theta +2\mathrm{bcos}}^{2}\theta \cos2\varphi )\;j_{y}=\mathrm{bsin}2\theta \sin2\varphi$$

where φ is the angle between the *y*-axis and the plane passing through the wave vector of the excitation light and its polarization vector, θ is the angle of incidence of the light, a and b are optical constants.

From Equation (3), it follows that the dependence of the photocurrent on the angle of rotation of the polarization vector of light is sinusoidal. For the longitudinal component of the photocurrent (leading to the generation of the TM component of the THz field), the photocurrent angle dependence is proportional to cos2φ. While for the transverse component of the photocurrent (leading to the generation of TE-components of the THz field), the photocurrent varies as sin2φ with the angle (see Figure 4). Exactly this behavior is observed experimentally (see Figure 7). In addition, in the presented formalism, it is expected that the transverse photocurrent is zero for excitation light of both TM and TE polarizations, which is also consistent with experiments. Therefore, our combined experimental results indicate that the mechanisms for photocurrent generation in α-Sn/Ge quantum well heterostructures are quite unusual. At the microscopic level, the nature of the processes for THz radiation generation in the α-Sn/Ge quantum wells samples may be due to the appearance of asymmetry in the momentum space arising from nonequilibrium electronic interband transitions, induced by the photon drag currents.

In addition, the significant increase in the efficiency of THz radiation generation in samples with Ge—α-Sn quantum wells may be related to an increase in the mobility of charge carriers $\mu =2500\pm 100\;{\mathrm{cm}}^{2}{\;V}^{-1}{\;s}^{-1}$. Moreover, the metallic behaviors of the temperature dependence of the resistivity in α-Sn/Ge quantum well heterostructures indicate a substantially different electronic system compared to pure Ge thin films and conventional semiconductors. The electronic band structures found in α-Sn/Ge quantum wells may contain a linear spectrum of two-dimensional electronic dispersion with massless charge carriers forming at the interface between Ge and α-Sn [4]. It is interesting that in an analogous system with a linear Dirac spectrum such as epitaxial graphene grown on 6H-SiC (0001) there are also remarkable photo-responses [31]. There the resistance shows also logarithmic temperature dependences which may be attributed to an Altshuler–Aronov effect. Note that the effect can be further enhanced by interface roughness or superlattice design, as noted for the harmonic conversion efficiency in semiconductor superlattices [32,33,34] or in graphene chips [35,36]. However, further in-depth studies are required to properly determine the mechanisms for the observed effects and to ascertain the role of α-Sn and topologically inverted band structures for THz generation and photocurrents.

## 3. Conclusions

In summary, we have prepared α-Sn/Ge quantum well heterostructures and confirmed the presence of α-Sn phase via Raman spectroscopy temperature dependence of the electrical resistivity. We have investigated the photocurrent and THz radiation generation in the α-Sn/Ge quantum well heterostructures. Our findings demonstrate that the mechanism for photocurrent generation in such systems is substantially different to those found in conventional thin-film semiconductors. We show that the angular dependence of the photocurrents (as well as the THz pulse amplitudes) may be explained by a photon drag current model. At the microscopic level, the origin of the THz radiation generation in this material may be linked to nonequilibrium electronic interband transitions induced by photon drag currents. These processes generate an asymmetry in phase space, which, combined with linear two-dimensional band dispersion and the mutually inverted band structure arising at the α-Sn/Ge interface, may acquire a topological character. Therefore, we postulate that the α-Sn/Ge quantum well heterostructures possibly represent a new kind of two-dimensional electronic system, which may give foundation to a novel type of two-dimensional topological insulator. However, further experimental and theoretical investigations are needed to properly clarify the underlying effects that determine the electronic properties in such systems.
